# Supplementary figures and images for: Sterol 14-α-demethylase is vital for mitochondrial functions and stress tolerance in Leishmania major
Source: PLoS Pathog. 2020 Aug 20;16(8):e1008810. doi: 10.1371/journal.ppat.1008810 (PMC7462297; doi:10.1371/journal.ppat.1008810)

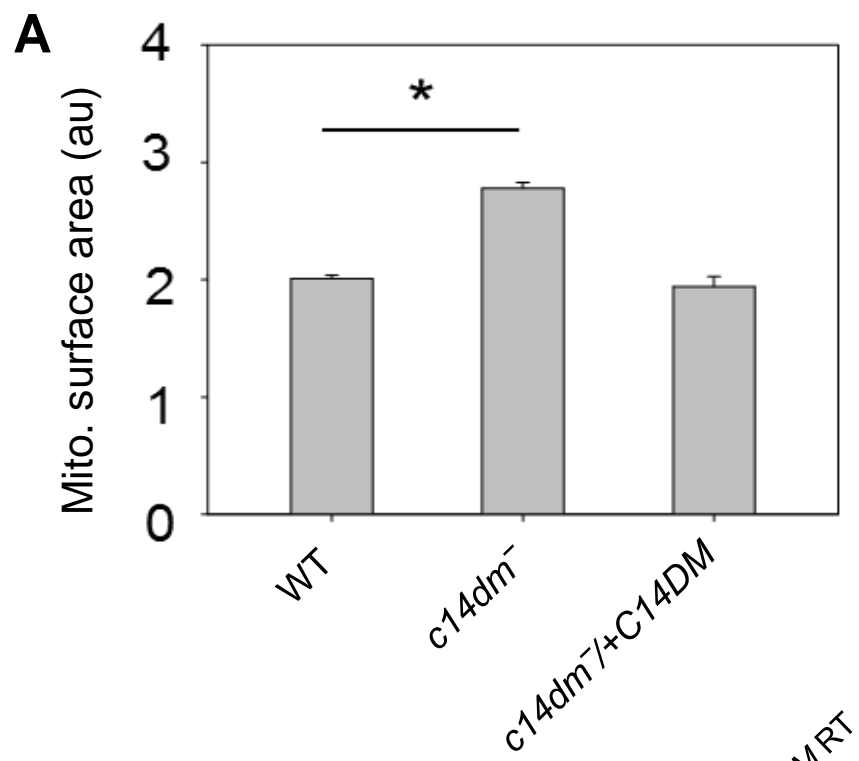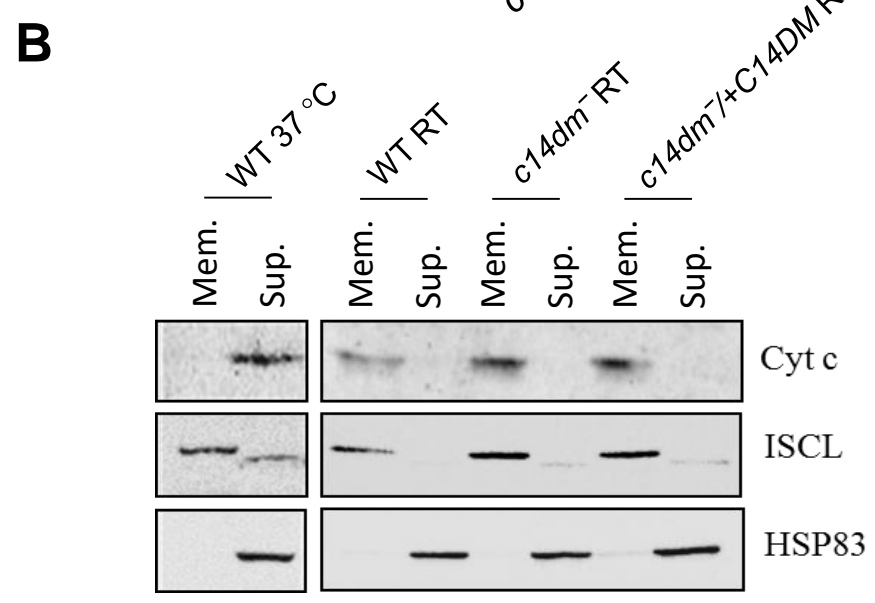

Supplement: S1 Fig — (A) After Mitotracker staining (Fig 1A), the average mitochondrial surface areas in log phase promastigotes of WT, c14dm¯, and c14dm¯/+C14DM were determined using Image J (~200 cells were analyzed for each parasite line, au: arbitrary unit). (B) Log phase promastigotes were lysed with digitonin at 37 °C or room temperature (RT) and mitochondria enriched membrane fractions (Mem.) were separated from cytosolic fractions (Sup.) as described in Materials and Methods. Western blots were performed using antibodies against cytochrome c, ISCL (a mitochondrial membrane protein), and HSP83 (a cytosolic protein). (PDF) [file ppat.1008810.s001.pdf]

**A**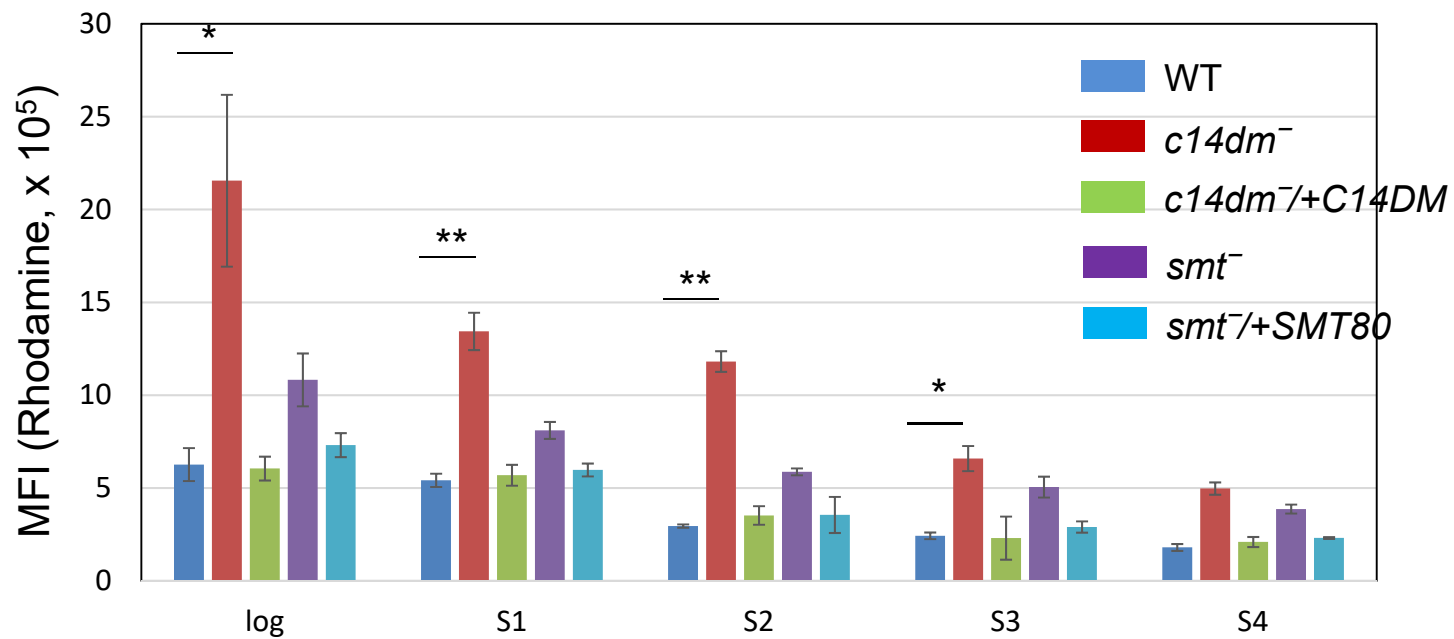**B**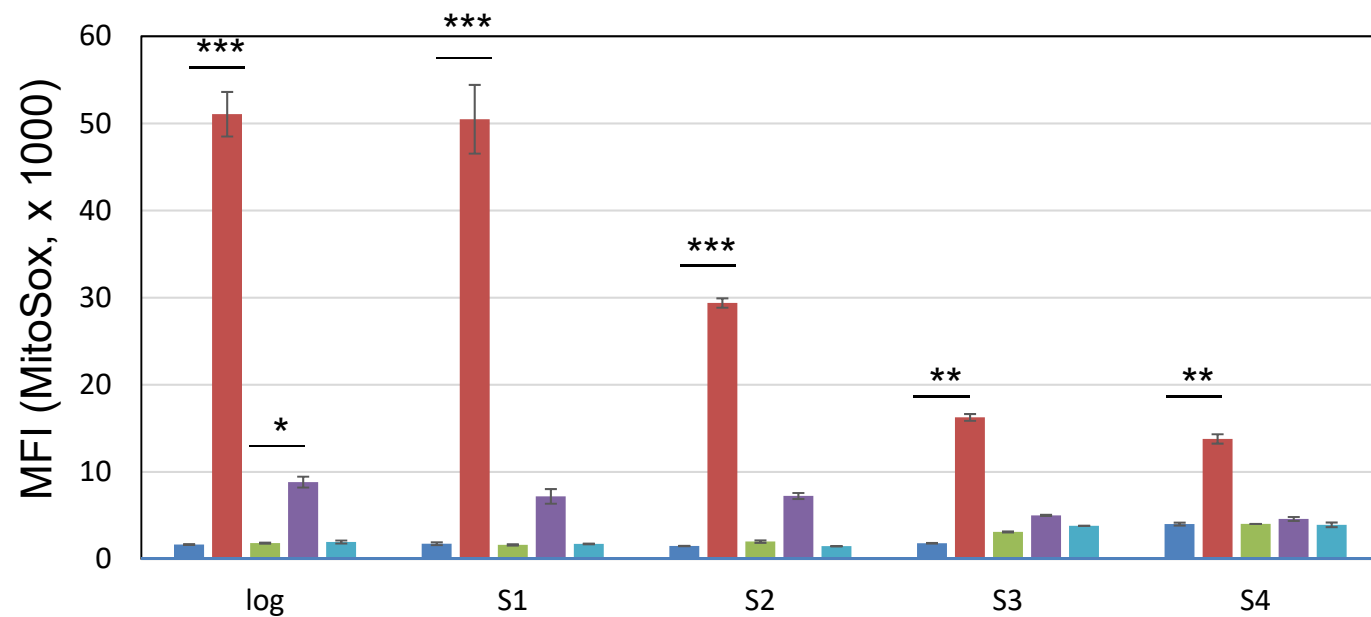

Supplement: S2 Fig — Log phase and stationary phase (day 1-day 4) promastigotes were resuspended in PBS and labeled with 5 μg/ml of rhodamine 123 for 15 min (A) or with 5 μM of MitoSox Red for 25 min (B) at room temperature. MFIs were determined by flow cytometry. Error bars represent standard deviations from three independent experiments. (PDF) [file ppat.1008810.s002.pdf]

**A**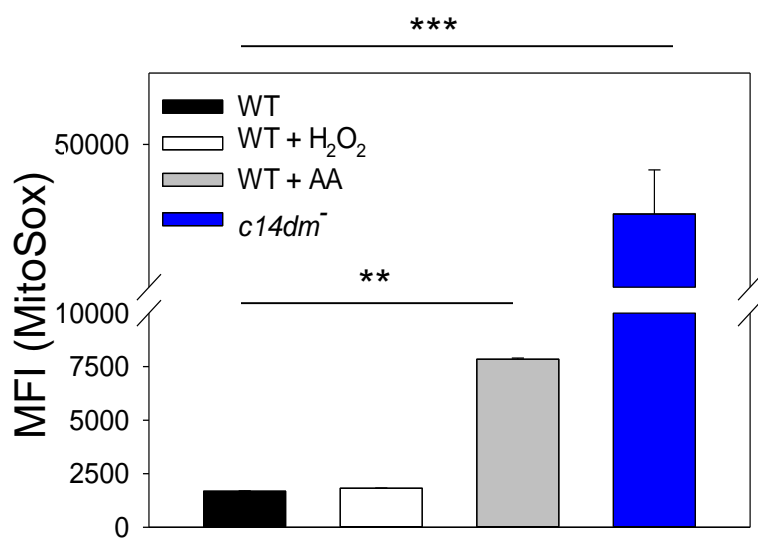**B**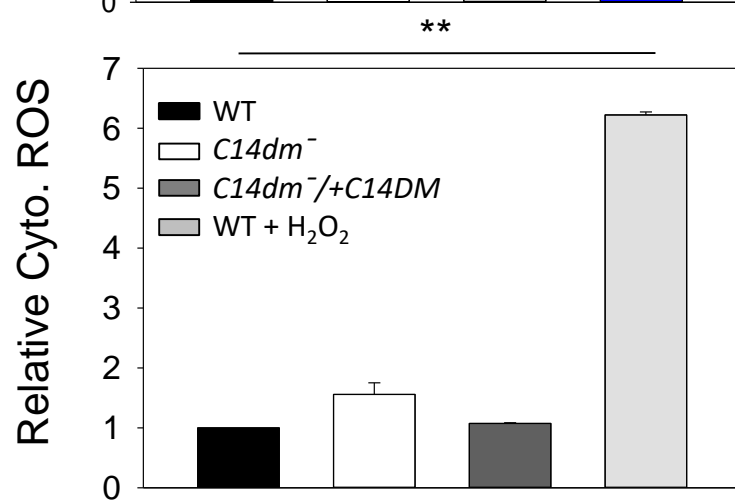

Supplement: S3 Fig — Log phase promastigotes were labeled with 5 μM of MitoSox Red for 25 min (A) or 5 μM of DHE for 30 min (B) and MFIs were determined by flow cytometry. Effects of AA (5 μM) and H2O2 (100 μM) on WT parasites were also monitored. Error bars represent standard deviations from three experiments. (PDF) [file ppat.1008810.s003.pdf]

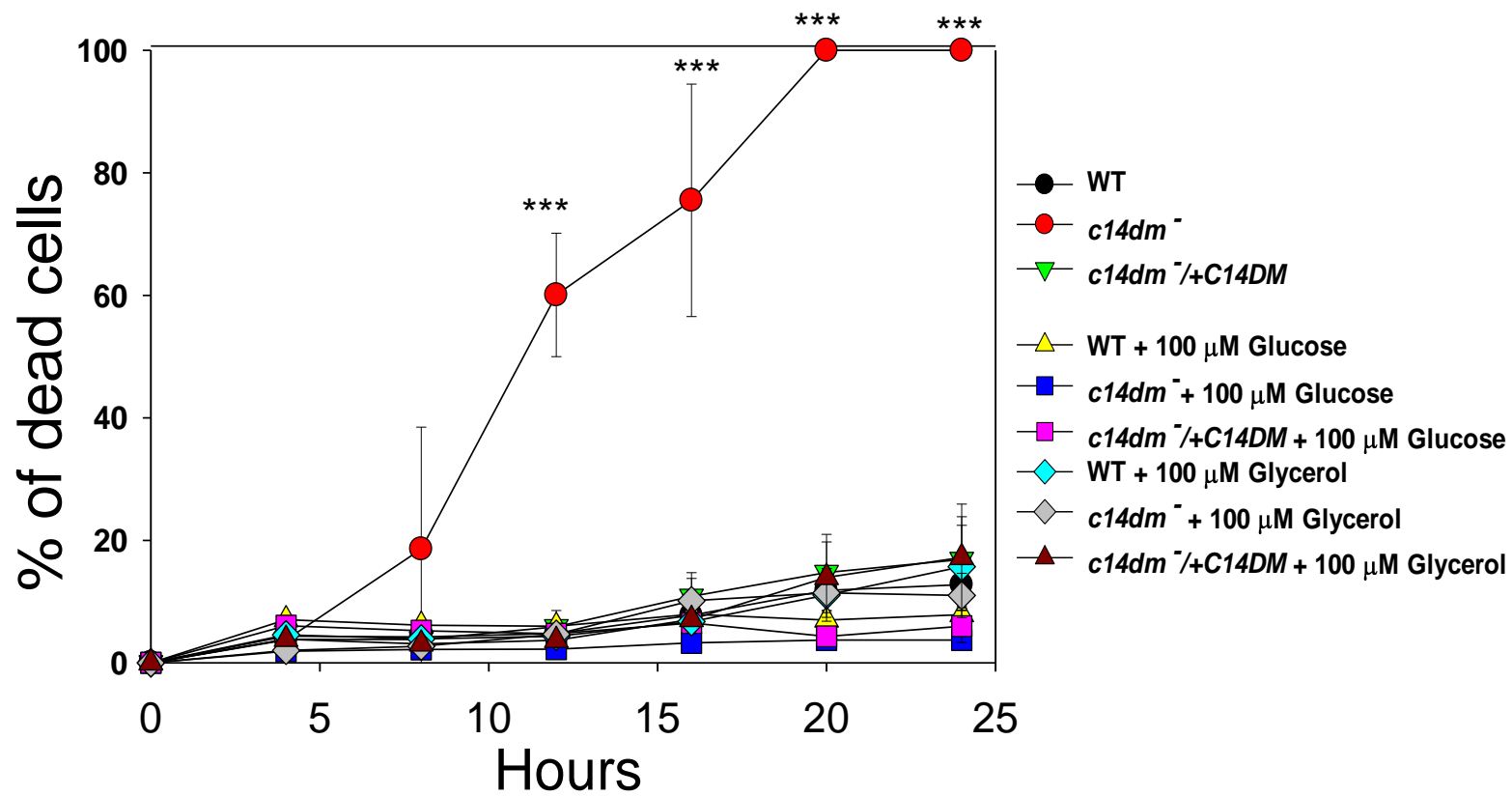

Supplement: S4 Fig — Log phase promastigotes were incubated in PBS in the absence or presence of glucose (100 μM) or glycerol (100 μM) and percentages of dead cells were determined by flow cytometry at the indicated time points. Error bars represent standard deviations from three experiments. (PDF) [file ppat.1008810.s004.pdf]

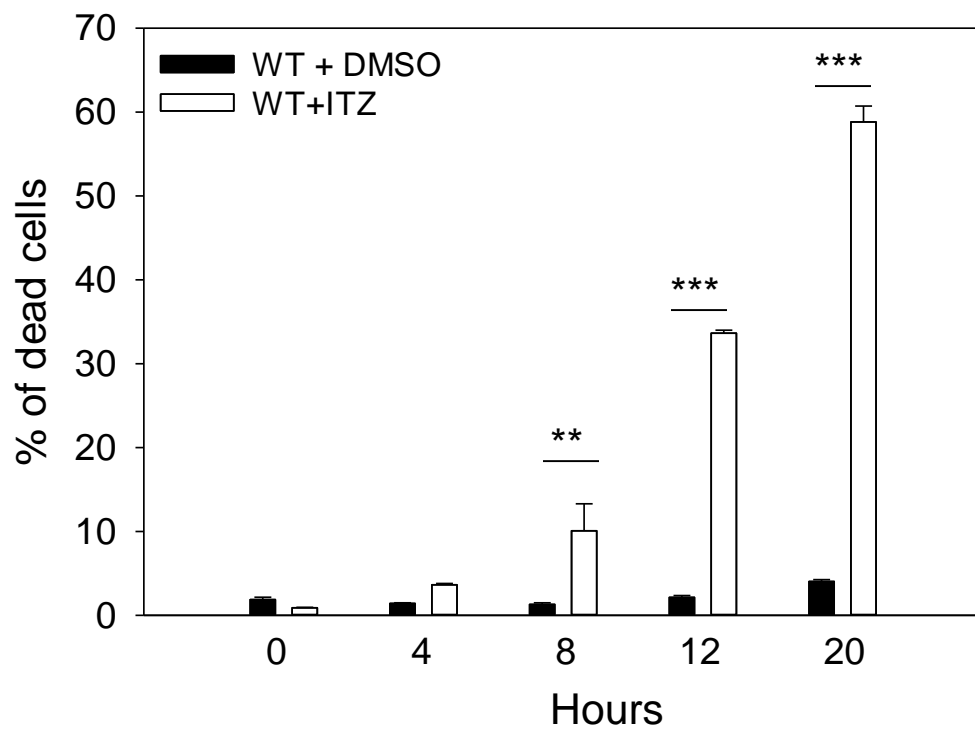

Supplement: S5 Fig — L. major WT promastigotes were cultivated in solvent alone (0.1% w/v of DMSO) or 0.2 μM of ITZ for 48 hours. Cells were then incubated in HBSS and percentages of dead cells were determined by flow cytometry at the indicated time points. Error bars represent standard deviations from three experiments. (PDF) [file ppat.1008810.s005.pdf]

**A**

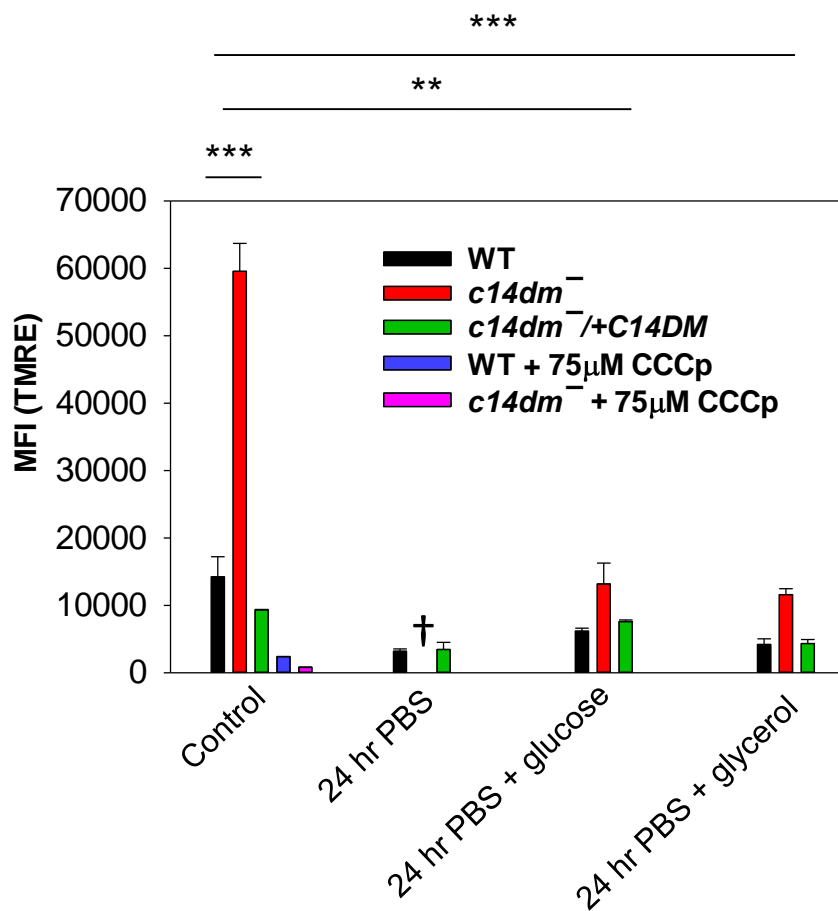

**B**

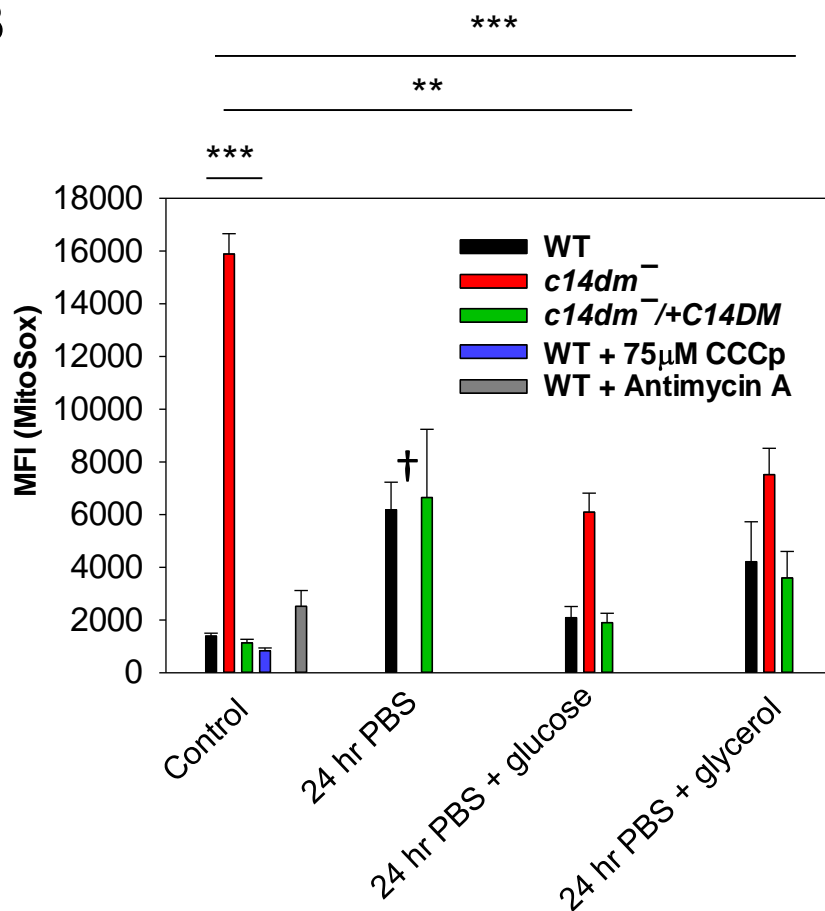

Supplement: S6 Fig — Log phase promastigotes were incubated in PBS in the absence or presence of glucose (100 μM) or glycerol (100 μM) for 24 hours. ΔΨm (A) and mitochondrial ROS level (B) were determined by flow cytometry. Control cells represent cells analyzed at the beginning of incubation. †: no viable c14dm‾ mutants were available after 24 hours. Error bars represent standard deviations from three experiments. (PDF) [file ppat.1008810.s006.pdf]
