# Supplementary material for: Sterol 14-α-demethylase is vital for mitochondrial functions and stress tolerance in Leishmania major
Source: PLoS Pathog. 2020 Aug 20;16(8):e1008810. doi: 10.1371/journal.ppat.1008810 (PMC7462297; doi:10.1371/journal.ppat.1008810)
Supplement: Attachment — Submitted filename: Response to review.docx [file ppat.1008810.s007.docx]

We thank the editor and reviewers for their insightful comments. We are also grateful to the editorial office for giving us additional time to complete our revision due to COVID-19. In this revision, we have provided new data (Figs. 4, 8, and S2) and modified the text to address the critiques from the editor and reviewers. Our point-by-point response is provided below with the editor’/reviewers’ comments italicized between quotation markers.

“*All referees considered this to be a solid piece of experimental work. However, views on acceptability for publication in Plos Pathogens were divided. Reviewer 1, in particular, felt that the gain in knowledge was incremental given that others have already identified roles of sterols in mitochondrial membrane composition and the current work is an additional piece of phenotypic analysis of the mutant line first published in Plos Pathogens in 2014. However, as I have an interest in this field I was particularly taken with the observation that the C14DM mutant has a substantially increased mitochondrial membrane potential. We have developed a series of amphotericin B resistant lines (L. mexicana) (PMID: 30716073 & PMID: 28622334). They have mutations in different sterol pathway enzymes, converging on a loss of ergostane type sterols. We find hypersenstivity to pentamidine a common feature in these parasites (and others unpublished). Since pentamidine's activity has some dependence on mitochondrial membrane potential and accumulation into that organelle (PMID: 12435669), the findings here offer a potentially satisfying explanation to this observation. You have previously shown the mutant is resistant to Amphotericin B. It would be useful to include data here on sensitivity to pentamidine to which they are likely to be hypersensitive). This would be a simple experiment.*”

We thank the editor for raising this intriguing point. Indeed, the *c14dm*‾ mutants (and WT parasites grown in the presence of ITZ) were hypersensitive to pentamidine (Fig. 8A, line 328-339), suggesting that the high mitochondrial potential and/or ROS make *c14dm*‾ vulnerable to agents that can exploit the mitochondrial defects such as pentamidine.

“*It would be further interesting to see if you could work out how long parasites need to be exposed to pentamidine (in wash out experiments) before they commit to death. If, as is true for African trypanosomes, fairly short exposures are needed (does dependent) it would be possible to set up experiments to see if transiently diminishing mitochondrial potential with CCCP could alter time dependence of pentamidine exposure to commit to death. Similar work was done with T. brucei as a possible marker on how to design experiments (PMID: 8103625).”

“If you can place your work in a more translational context of chemotherapeutic relevance I feel the work's significance will be greatly enhanced.*”

As suggested by the editor, we performed the pentamidine wash out experiment (one hour incubation in 2.5 µM or 25 µM of pentamidine followed by washes and re-inoculation in drug-free M199 media (Fig. 8B-C). Clearly, 2.5 µM pentamidine exposure led to significant grow delay in *c14dm*‾ but not WT or add-back parasites. The impact was much more significant at 25 µM. Treatment with CCCp alleviated the sensitivity in *c14dm*‾, suggesting that increased mitochondrial potential and/or ROS enhanced the cytotoxic effects of pentamidine (Fig. 8B-C).

We agree that these new findings allude to the potential of exploiting the fitness loss associated with drug resistance (e.g. to amphotericin B and azoles), thus enhance the translational significance of this study.

“*A few additional comments I'd make, in addition to those of the other reviewers:*”

“*Line 14 - mitochondrial not mitochondria*”

We have changed the short title to “Sterol synthesis affects mitochondria in *Leishmania*”.

“*Line 68 - can a comment on amphotericin B activity in amastigotes be included given their apparent low level of ergostanes*?”

Amphotericin B binds to ergosterol-like lipids leading to pore formation and ROS accumulation so in theory, even minute amount of endogenous sterol can lead to amphotericin B sensitivity. In addition, ergostane-based sterols (even at low levels) may be vital for the plasma membrane and/or organelle membrane functions in amastigotes (line 67-70).

‘*Line 109 "quite unique" is not really corerct, either in the meaning of the word "unique" not in biological relevance. Lots of organisms have a single mitochondrion, so just drop the "quite unique"*’

This has been corrected.

‘*Line 140: add "at least with regard to cytochrome c localisation between "integrity" and "under" (other aspects of the membrane's integrity weren't looked at)*’

This has been corrected.

“*Line 327: also in considering more pharmacological relevance in a revised manuscript - could triazole/pentamidine combinations be considered (assuming you find pentamidine hypersensitivity)*”

We have included WT + ITZ in the pentamidine experiments (Fig. 8) and provided such comments (line 328-351, 428-435 and 444-447).

“*REFERENCES: Please check the format of each against Journal requirements. Note that reference 2. uses abbreviated journal title name, others use whole title, so check for consistency.*”

This has been corrected.

Reviewer's Responses to Questions

**Part I - Summary**
Please use this section to discuss strengths/weaknesses of study, novelty/significance, general execution and scholarship.

“*Reviewer #1: This article by Mukherjee et al. investigates the role of the sterol biosynthetic enzyme C14-α-demethylase (C14DM) in mitochondrial function in promastigotes of Leishmania major. This enzyme is of interest, because it is the target of azole compounds that have efficacy as antifungals and have been investigated as potential anti-leishmanial drugs. In previous studies, the authors have generated the c14dm- null mutant and shown that plasma membrane fluidity is decreased in this mutant, that it is susceptible to increased temperature, and that its virulence in mice is greatly reduced. Of interest, these effects are probably due to accumulation of C14-methylated sterol precursors when the demethylase is knocked out or inhibited rather than to the absence of the mature sterol. The current study focuses on the effects of the c14dm- mutation, and of treatment with the C14DM inhibitor itraconazole (ITZ), on the mitochondrion. Although most sterol end products in eukaryotic cells (e.g., cholesterol or ergosterol) are present in the plasma membrane or vesicles that traffic to the PM, sterols are also present in organellar membranes including the mitochondrion, albeit at lower levels.”

“Indeed, the authors find major alterations in promastigote mitochondria in the c14dm- mutant and in parasites treated with ITZ. Mitochondria increase in size, the mitochondrial membrane potential is significantly increased, and levels of reactive oxygen species (ROS) increase by 10-15-fold in the mitochondrion, probably due to leakage of electrons from the mitochondrial electron transport chain due to alterations in the inner mitochondrial membrane. Furthermore, oxygen consumption is reduced in the c14dm- mutants, and the parasites appear to switch to greater reliance upon glycolysis for generation of cellular ATP. The promastigotes thus become highly sensitive to glucose restriction, but supplementation of the medium with either glucose or glycerol protects the parasites against loss of viability. Furthermore, shifting temperature from 27° to 37° also induces mitochondrial alterations in these mutants, including reduced membrane potential, leakage of cytochrome oxidase out of the mitochondrion, and increased cytosolic ROS, leading to cell death. Overall, these studies implicate a critical role for sterols in maintenance of mitochondrial function in these parasites and imply that azoles may function, at least in part, by impairing mitochondrial function.”

“This is a well-executed study that employs a variety of assays to assess mitochondrial function in wild type, c14dm- mutant, and ITZ-treated promastigotes. Appropriate scientific rigor is applied, with multiple replicates and statistical analysis of data. The paper reports a substantial body of work, and the studies should be of interest to the molecular parasitology community. The major new material is the elucidation of effects of blocked sterol biosynthesis on mitochondria, as opposed to earlier studies by this group that focused upon the plasma membrane, the principal site of sterol accumulation. Nonetheless, the novelty is probably not exceptional. The c14dm- mutant has been investigated previously and shown to have significant deficiencies, including on virulence. Sterols have been known to partition into intracellular organelles for some time, as indicated in various review articles published over more than a decade. Previous studies on trypanosomatids, reviewed by these authors on pages 5-6 of this manuscript, have implicated the mitochondrion in impairments induced by azoles, although they have not been as detailed or definitive as the current work. Hence, this is a strong study focused on a pathway of interest for possible drug development, but perhaps not of exceptional novelty or interest.*”

“*Reviewer #2: In this manuscript, Mukherjee et al. have explored the impact of genetic or pharmacological ablation of sterol 14α-demethylase (c14dm-) activity on the function and integrity of the mitochondrion in Leishmania major. Previous studies have demonstrated a link between disruption of sterol biosynthesis and the mitochondrion in both Leishmania and Trypanosoma, however, these studies were observational and lacked the systematic evaluation offered in this manuscript. The authors, through a meticulous set of experiments, that were appropriately controlled and which offered clear outcomes to testable hypotheses, have demonstrated that disruption of C14DM leads to the interruption of energy metabolism in the mitochondrion, making these cells highly dependent on glycolysis to meet their energy needs. The dysfunction in the mitochondrion appears to be linked to an impairment in ATP synthesis via ATP synthase, which leads to an increase in mitochondrial membrane potential and ROS production – the likely cause of the increased sensitivity of these c14dm- cells to stress.”

“The quality of the work in this paper is exceptionally high, the results unambiguous, and the work reaffirms C14DM, and indeed the sterol biosynthetic pathway, as an important drug target in these parasites worthy of further exploration. The work expands our current knowledge of the mechanisms of action of the azole drugs and, since it is highly likely it can be extrapolated to other trypanosomatids, should be of broad interest to the field. I have no suggested major revisions, and only a few minor comments to improve the overall manuscript.*”

“*Reviewer #3: This manuscript builds on the authors’ prior work on endogenous sterol synthesis in Leishmania major and the implications for drug design. The manuscript is clearly written and cites the existing literature well. A major strength of work from this group (including this manuscript) is the use of genetic knockout mutants (c14dm-/- and smt-/-) to understand parasite biology instead of the sole use of chemical inhibitors of these enzymes that have potential for off target impacts. The authors state that further characterization of sterol synthesis in the clinically relevant stages of Leishmania would provide interesting and potentially different insights. It is a point well made by the authors but may complicate some of the stated significance (Line 19). Some of the conclusions with regard to “adaptive” responses are overstated and not in line with the data.*”

Thanks for the assessments. In light of the fact that *c14dm*‾ mutants (and WT parasites grown in the presence of ITZ) show hypersensitivity to pentamidine, our study suggests that combinations of ITZ + pentamidine or amphotericin B + pentamidine can yield synergistic effects or prevent the emergence of drug resistance. As commented by the editor, the new findings provide more translational relevance for this study. While our studies were performed on promastigotes, both amphotericin B and pentamidine have shown efficacy against leishmaniasis, suggesting that such combination can be effective against amastigotes. We have revised the text that may have over-interpreted the data.

**Part II – Major Issues: Key Experiments Required for Acceptance**
Please use this section to detail the key new experiments or modifications of existing experiments that should be absolutely required to validate study conclusions.

Generally, there should be no more than 3 such required experiments or major modifications for a "Major Revision" recommendation. If more than 3 experiments are necessary to validate the study conclusions, then you are encouraged to recommend "Reject".

Reviewer #1: (No Response)

Reviewer #2: None suggested

Reviewer #3: As written, some of the conclusions regarding the relative roles of glycolysis and mitochondrial respiration in the c14dm-/- mutants vs WT parasites are a bit misleading (Fig 4).

“*Figure 4A: The graph shows that the c14dm-/- mutant exhibits reduced mitochondrial respiration as compared to the WT and add-back parasites. But what is presented are not the basal respiration rates for each line and therefore not a fair initial comparison. In this experiment, OCR was measured in the presence of 2-DG (line 478) to block glucose utilization – and because there is a bigger effect on the mutant, it appears as if respiration in the mutant line is impaired under basal conditions, which may or may not be the case. Therefore, a figure should be included to show basal respiration rate (OCR) measured in log-phase c14dm-/- mutants as compared to WT and add-back parasites incubated with a full complement of substrates. This data would determine if the mitochondrial defects observed (e.g. Fig1/2) result in defects in respiration. Based on the buffer composition described for Fig. 4 the OCR data appear to be oxygen consumption solely from pyruvate. These conditions, and the inferences gained need to be clarified in the text.*”

“*The authors should include the raw MitoXpress data for these experiments - linear plots as supplemental data. Also, the OCR value should be normalized to parasite number or protein in each sample.*”

We now included the raw OCR data for log phase parasites in respiration buffer (HBSS with sodium pyruvate and 2-DG) and DMEM in Fig. 4A and Fig. 4C respectively. When parasites were incubated in DMEM (containing 4.5 g/ml or 25 mM of glucose), their OCRs were much higher than those in the respiration buffer (Fig. 4A and C). We were unable to reach the maximal reading for WT in Fig. 4C due to signal saturation in the BioTek synergy 4 fluorescence microplate reader. Under both nutrient replete and nutrient limiting conditions, *c14dm*‾ mutants showed lower OCR than WT and add-back, suggesting that their mitochondrial defects led to respiration deficiency (Fig. 4A-C).

Due to the lack of a calibration curve with this MitoXpress approach, we could not determine the actual OCR in pmol/μg of protein/min or pmol/10^6^ cells/min. For each cell line, the relative OCR in respiration buffer was calculated by subtracting the 0 min reading from 15 min reading (Fig. 4B). At 15 min, cells had yet to reach their maximal oxygen consumption (Fig. 4A). For this assay, 2 x 10^6^ cells in 100 μl oφ respiration buffer were used for each cell line (line 513-527).

“*Secondly, based on results presented in Fig 4, the authors emphasize increased dependence of the c14dm-/- mutant on glycolysis which might be expected because of the ‘mitochondrial defects’ observed in this mutant. However, as shown in Fig. 4B, most of the ATP production in the c14dm-/- mutant comes from mitochondrial respiration, not glycolysis. While glycolysis might contribute a bit more to the ATP produced by the mutant than the WT, it appears to be a minor contribution. Moreover, the authors do not include data to confirm the presumptive contribution of glycolysis to the residual ATP produced by the mutant in the presence of sodium azide. Data should be included to show that this signal is completely ablated if an excess of 2-DG is added to block glycolysis.”*

*“It is unclear why the buffer used in Fig. 4B (line 486) contains 2-DG. The figure legend does not state that 2-DG is in the buffer and may confuse readers. How is ATP production is expected to be altered in the absence of 2-DG in the buffer?*”

“*Also, in Fig 4B, in the absence of an exogenous carbon source (for 1 hr), there was no difference in the amount of ATP produced by the c14dm-/- mutants, WT and add-back. What is the major contributor to this basal ATP production – glycolysis, mitochondrial respiration? Does sodium azide or 2-DG treatment differentially impact the mutants and WT parasites? What does the pattern look like when parasites have access to a mixture of usable carbons (and absence of 2-DG in the buffer)? This information should be included and results interpreted in context.*”

To clarify the ATP production in *c14dm*‾ mutants, we repeated the 1-hour assay (line 528-536) using log phase promastigotes incubated in a neutral, isotonic buffer without carbon source (Fig. 4D, lane 1-3), with 5.5 mM of glucose (Fig. 4D, lane 4-6), or with 5.5 mM of sodium pyruvate (Fig. 4D, lane 7-9). In each set, 2-DG (5.5 mM) or sodium azide (20 mM) were provided as indicated in Fig. 4D. Averages from 4 independent repeats were summarized in Fig. 4D.

Without additional carbon source, *c14dm‾* mutants had 30-40% more ATP than WT or add-back parasites (Fig 4D, lane 1) despite having a lower OCR (Fig. 4A-B). Supplementation with glucose led to increased ATP production in all cells and *c14dm‾* mutants again produced 30-40% more ATP than WT and add-back parasites (Fig 4D, lane 4) while having a lower OCR (Fig. 4C). Thus, we postulate *c14dm‾* mutants have increased capacity to use glycolysis to generate ATP. As expected, 2-DG (5.5 mM) significantly reduced ATP production in the absence of exogenous glucose (Fig 4D, lane 2 and 8). No significant reduction was detected when cells were incubated in the presence of glucose (5.5 mM), which was likely due to inadequate competition on glucose uptake and utilization (Fig 4D, lane 5). Addition of sodium azide drastically lowered ATP production in all cells (Fig 4D, lane 3, 6 and 9), suggesting that the glycolysis flux may be coupled to the efficiency of oxidative phosphorylation respiration in *Leishmania* so that cells can downregulate glycolysis to prevent the buildup of toxic metabolites (line 238-257).

“*When glucose is the only available exogenous substrate, the c14dm-/- parasites produce more ATP than WT parasites (there is no significant difference between mutant and add-back parasites) and the mutants take up more glucose and glycerol than the WT or add-back parasites. These data support the idea that the mutants might rely more heavily on glucose than other substrates to fuel energy production. Exogenous pyruvate or aspartate fail to rescue mutants from glucose starvation. In this scenario, is it possible to rule out impaired plasma membrane or mitochondrial permeability to other potential TCA substrates in the c14dm-/- mutants?*”

“*Along the same line, in some experiments, pyruvate is included in the parasite incubation buffer – presumably to fuel the TCA cycle and mitochondrial respiration. It doesn't seem like the inclusion of pyruvate makes a difference in the ability of WT parasites to generate ATP. The amount of ATP produced by all parasite lines in HBSS alone (Fig 4B, first set of bars) and HBSS + pyruvate present (Fig. 4C, first set of bars) appears to be similar. Why doesn’t ATP production increase in the presence of exogenous pyruvate and what is the effect on OCR?*”

“*At 4 hr in the absence of glucose (Fig4C) – but in the presence of pyruvate - the c14dm-/- mutants start to lose the capacity for ATP synthesis. Given that mitochondrial respiration is the main source of ATP in the c14dm-/- mutants (Fig. 4B), exogenous pyruvate should sustain ATP production at a higher level in the mutant if it is being utilized. Is ATP production lost at a higher rate in the mutant in the absence of exogenous pyruvate?*”

“*Has it been demonstrated that pyruvate is transported across the parasite plasma membrane so that it can be utilized by the mitochondria? In order to draw conclusions about substrate utilization and mitochondrial function, it is important to demonstrate that these important assumptions of the assay (e.g. substrate transport over the plasma membrane) are verified. Some of these data can be explain through substrate permeability differences between parasites.*”

Yes in our whole cell experiments, addition of sodium pyruvate had little effect on the cellular ATP contents (Fig 4D, lane 7). Although *Leishmania* parasites possess strong, syntenic orthologues to the plasma membrane-bound pyruvate transporters in *T. brucei* (Sanchez MA, 2013, reference [48]), the efficiency of pyruvate uptake is not known. So, we cannot rule out the possibility that *c14dm‾* mutants have impaired plasma membrane or mitochondrial permeability to pyruvate. However, these mutants exhibit increased mitochondrial membrane potential and ROS (Fig. 1-2), which suggest ETC defects rather than substrates permeability defects. Another possibility is that parasites can sense the lack of carbon sources besides pyruvate and regulate ATP biogenesis accordingly. We did not include the 4-hour experiment in the revised manuscript which showed similar results as the 1-hour assay.

**Part III – Minor Issues: Editorial and Data Presentation Modifications**
Please use this section for editorial suggestions as well as relatively minor modifications of existing data that would enhance clarity.

Reviewer #1: Minor Points.
“*I would recommend changing the order of Figs. 5A and 5B, as the latter panel is introduced first in the manuscript.*”

The order of Fig. 5A and 5B has been switched.

Reviewer #2: “• *The error bars on Fig. 4B are very faint and need to be made more visible.*”

This has been changed.

*“• The method used for assessing cell death by propidium iodide staining, though standard, is not detailed or referenced in the methods.”*

The information has been provided (line 465-467).

*“• There are few grammatical/typographical errors throughout the manuscript that need to be addressed prior to publication.”*

We have carefully proofread the manuscript and corrected the errors.

*“• Not a revision, but a question for the authors consideration. I’m curious if the increased uptake of glycerol is related to the enhanced activity of AQP1 and if so, how that might affect the sensitivity of the c14dm- cells to antimony drugs. Is something that is known or that has been tested?*”

Thanks for the suggestion. We are in the process of assessing the plasma membrane permeability of *c14dm‾* to hydrophilic and hydrophobic compounds. Examination of antimony uptake/sensitivity in *c14dm‾* may provide more significance to that ongoing study like the pentamidine work to this manuscript.

Reviewer #3: “*Line 66-68. What is the evidence in [7] that scavenging predominates and there is a downregulation of ergosterol synthesis? If this is true what is the cause of the attenuated growth in vivo of the mutants (i.e. would there be a reduction in 14-methylated sterols?)? In ref 7 the statement “Clearly, some of the cholesterol was not directly associated with amastigotes instead from mouse cells” suggests that the extent/importance of cholesterol scavenging is not entirely settled with regard to amastigotes.*”

GC-MS analysis of lesion-derived amastigotes in reference [7] (Figures S14 and S15 in the supporting information) demonstrate that the vast majority of sterols in WT and *c14dm‾* amastigotes is cholesterol (salvaged from the host). The abundance of parasite sterols, i.e. ergostane-based sterols in WT amastigotes and 14-methylated sterols in *c14dm‾* amastigotes are much lower by comparison. Because amastigotes were isolated from infected mouse tissue, it is very difficult to remove all the contaminants from mice which also contain cholesterol. Nonetheless, microscopy analysis indicate that the amastigote preparation was highly enriched for amastigotes and largely devoid of contaminants (Figure S13 in reference [7]).

In addition, 11 of 19 sterol biosynthetic genes are downregulated at the transcript level during the amastigote stage (tritrypdb.org). These findings strongly argue that the slow growing intracellular amastigotes acquire the majority of their sterols through salvage. Meanwhile, the residue amount of endogenous sterols may play vital roles in amastigotes which constitutes the basis for sterol biosynthetic inhibitors as anti-leishmaniasis drugs. Future studies will elucidate how intracellular parasites acquire their lipids and the contribution of amastigote lipids to their survival.
“*Fig 3B and line 210-211. Without a loading control or quantification the claim of “similar” seems to be a fair characterization but “slightly higher” may be a stretch.*”

This has been corrected (line 215-216).

“*Line 216: The statement “..outside of SOD deficiency.” might be interpreted, as SOD is sufficient to control ROS (which the data suggest it may not be). Would it be fair to state that parasite SODs appear unaffected but are apparently unable to alleviate excess ROS production?*”

Agreed. We have modified the statement (line 220-221).

“*Line 447 “Such treatments did not affect parasite viability based on PI-staining.” It is unclear if PI was used for all of the flow experiments to verify the integrity of the parasites. If this is the case it would be worth mentioning in the results section to support the conclusions when treatments like CCCP lead to a negative result (i.e. Fig 2A/B). This would further strengthen the authors conclusions as opposed to any results being due to alterations of general viability. Additionally (but unlikely under the stated treatment times) parasite death leading to lysis will not lead to a detectable event (PI pos or negative) to establish viability. Am I correct in assuming that parasites are not PI positive and the number of events (i.e. parasites) is consistent between treatment groups?*”

Yes we verified cell viability with PI staining in all ΔΨm and ROS measurements (20,000 events per sample in flow cytometry, line 154-155 and 485-489). The percentages of PI-positive were <5% unless otherwise specified. Addition of CCCp, antimycin A, or H_2_O_2_ at the indicated concentrations for 15-30 min did not cause significant cell lysis by our observation or by flow cytometry, as these compounds are not known to have detergent-like effects on cell membrane.

“*A major contribution of the generation of the smt-/- line (used in the 2018 publication) is the ability to compare phenotypes to the c14dm-/- line. This comparison (as noted by the authors) can help delineate if a given phenotype is caused by generation of 14-methylated sterols or absence on an endogenously synthesized sterol. In Figure 5A the smt-/- is used to support the notion that 14-methylated sterols contribute to the dependence on glucose. However, to support the notion that loss of mitochondrial membrane potential in the c14dm-/- is the cause of this glucose dependence it would be preferrable to see a side by side TMRE values (i.e. smt-/- and c14dm-/- ).”*

“*Lines 196-199 reference the 2018 publication as “not as profound” but a direct comparison/fold change would be more illustrative.*”

We have now included the *smt‾* results in the new Fig. S2 along with *c14dm‾* (line 191-194).
